# A transdiagnostic systematic review and meta-analysis of ketamine’s anxiolytic effects

**DOI:** 10.1177/02698811231161627

**Published:** 2023-04-02

**Authors:** Hannah Hartland, Kimia Mahdavi, Luke A Jelen, Rebecca Strawbridge, Allan H Young, Laith Alexander

**Affiliations:** 1 Department of Psychological Medicine, School of Academic Psychiatry, Institute of Psychiatry, Psychology and Neuroscience, King’s College London, London, UK; 2 South London and Maudsley NHS Foundation Trust, London, UK

**Keywords:** Ketamine, anxiety, anxiolytic, antidepressant, dissociation, meta-analysis

## Abstract

**Background:**

Ketamine may be effective in treating symptoms of anxiety, but the time profile of ketamine’s anxiolytic effect is ill-defined. This systematic review and meta-analysis investigated the anxiolytic effect of ketamine at different time points across a range of clinical settings.

**Methods:**

Electronic databases were searched to capture randomised control trials measuring the anxiolytic effects of ketamine in contexts including mood disorders, anxiety disorders and chronic pain. Meta-analyses were conducted using a random-effects model. The correlations between (1) improvements in mean anxiety and depression scores, and (2) peak dissociation and improvements in mean anxiety scores were also assessed.

**Results:**

In all, 14 studies met inclusion criteria. Risk of bias was high in 11 studies. Ketamine significantly reduced anxiety scores compared to placebo at acute (<12 h; standard mean difference (SMD): −1.17, 95% confidence interval (CI) [−1.89, −0.44], *p* < 0.01), subacute (24 h; SMD: −0.44, 95% CI [−0.65, −0.22], *p* < 0.01) and sustained (7–14 days; SMD: −0.40, 95% CI [−0.63, −0.17], *p* < 0.01) time points. Exploratory analyses revealed improvements in anxiety and depression symptoms correlated at both subacute (*R*^2^ = 0.621, *p* = 0.035) and sustained time points (*R*^2^ = 0.773, *p* = 0.021). The relationship between peak dissociation and improvement in anxiety was not significant.

**Conclusions:**

Ketamine appears to offer rapid and sustained anxiety symptom relief across a range of clinical settings, with anxiolytic effects occurring within the first 12 h of administration and remaining effective for 1–2 weeks. Future studies could explore the effects of ketamine maintenance therapy on anxiety symptoms.

## Introduction

Anxiety disorders are common, with a lifetime prevalence of 33.7% in US adults (Bandelow and Michaelis, 2015). The disorders share common features of fear and anxiety impairing daily function, but differ in the situations or objects catalysing the schema (American Psychiatric Association, 2013). Significant anxiety symptoms are seen in a range of clinical settings, including major depressive disorder (MDD) (Drysdale et al., 2017; Park and Kim, 2020), chronic pain (McWilliams et al., 2003), advanced cancer (Roth and Massie, 2007) and palliative care (Kelly et al., 2006; Kozlov et al., 2019; Neel et al., 2015).

Current treatments for pathological anxiety include selective serotonin reuptake inhibitors, but these have a slow onset of action and can paradoxically worsen anxiety upon initiation (Cassano et al., 2002; Nutt, 2005). Benzodiazepines provide rapid relief of acute anxiety symptoms, but are associated with tolerance and dependence (Bystritsky, 2006; Cassano et al., 2002). Given these issues and the prevalence of treatment-resistant anxiety (Bystritsky, 2006), there is a pressing need for the development of novel rapidly acting anxiolytic agents with the potential for intermediate to long-term administration.

One avenue which may meet this need comes is the N-methyl-D-aspartic acid (NMDA) receptor antagonist, ketamine. Ketamine is an effective glutamate-based antidepressant (Marcantoni et al., 2020), but evidence has also emerged supporting its anxiolytic effects. In patients with social anxiety disorder (SAD) (Glue et al., 2018; Taylor et al., 2018), obsessive-compulsive disorder (OCD) (Bandeira et al., 2022), post-traumatic stress disorder (PTSD) (Liriano et al., 2019) and treatment-refractory anxiety (Tully et al., 2022), ketamine may have fast-acting anxiolytic effects lasting approximately 1–2 weeks after a single dose.

Several review articles have explored ketamine’s utility in the treatment of anxiety disorders and have supported this suggestion (Banov et al., 2020; Tully et al., 2022; Whittaker et al., 2021). However, these reviews did not examine the time course of ketamine’s action, and included open-label studies, uncontrolled trials, case series, and other lower quality studies, limiting the conclusions that can be drawn about ketamine’s anxiolytic effects compared to placebo. Furthermore, the transdiagnostic nature of anxiety symptoms, as emphasised by the negative valence system in the *Research Domain Criteria* (Insel et al., 2010) and the fear and distress subfactors in the *Hierarchical Taxonomy of Psychopathology* (Kotov et al., 2017), means that ketamine may prove to be useful in a range of clinical settings associated with distressing anxiety symptoms and this has yet to be explored.

The purpose of this systematic review and meta-analysis is to examine the effect of ketamine on symptoms of anxiety at several time points, through synthesizing the findings of blinded, randomised, placebo-controlled trials (RCTs). Our principal analyses explored ketamine’s anxiolytic effects acutely (<12 h), subacutely (24 h) and at a sustained time point (7–14 days), incorporating data from RCTs measuring anxiety symptoms in anxiety disorders, depression (and other mood disorders), chronic pain, and palliative care settings.

## Methods

This review was carried out in accordance with PRISMA guidance. We registered the protocol for this review with the PROSPERO International prospective register of systematic reviews (CRD42022303070; URL). In addition to the protocol described on PROSPERO, we collected depression and dissociation data and correlated these against changes in anxiety scores as described in the section ‘Data extraction and analysis’.

### Inclusion and exclusion criteria

Our inclusion criteria were as follows:

- *Study type*: single- or double-blinded RCT written in English (including crossover trials);
- Population: adult human patients suffering from anxiety disorders of any type (including PTSD and OCD) or in whom anxiety symptoms were measured in the context of mood disorders, chronic pain or palliative care;
- Intervention: subanaesthetic doses of racemic ketamine, S-ketamine or R-ketamine, administered via intravenous, intranasal, oral, subcutaneous, intramuscular or sublingual routes;
- Control: an active or inactive placebo comparator;
- Outcome: a primary or secondary outcome relating to anxiety.

We excluded the following:

- Animal studies;
- Non-RCT studies or where no party was blinded to treatment allocation;
- Any studies where ketamine was used at anaesthetic doses or in the context of surgery/anaesthesia;
- Any studies where ketamine was administered in the context of a medical or surgical emergency;
- Studies with child or adolescent subjects;
- Studies where anxiety scores were not reported as a primary or secondary outcome.

### Search strategy

EMBASE (Ovid), MEDLINE (Ovid) and APA PsycINFO (Ovid) were systematically searched (February 2022) using keywords and medical subject headings relating to ketamine and anxiety: ketamine AND [(Anxiety OR Test Anxiety OR Anxiety Disorders OR Test Anxiety Scale) OR Obsessive-Compulsive Disorder OR Social Phobia OR Phobic Disorders OR Agoraphobia OR Post-Traumatic Stress Disorders OR Separation Anxiety OR (Panic Disorder OR Panic) OR Mutism OR (GAD-7 OR Patient Health Questionnaire) OR (Palliative Care OR ‘Hospice and Palliative Care Nursing’ OR Palliative Medicine)] AND (Randomized Controlled Trial OR Clinical Trial) NOT Child. In addition, the first 200 findings of a Google Scholar search (February 2022) using keywords (ketamine AND anxiety) were examined.

### Article screening and assessment for eligibility

Any duplicate titles and abstracts generated from the search were removed using Syras systematic review screening software (Scipilot Pty. Ltd., 2021, Sydney, Australia). The remaining articles were screened for potential relevance and eligibility according to pre-specified inclusion and exclusion criteria by three independent researchers, with others’ ratings masked from their own.

### Risk of bias

To determine reliability and transparency of the studies, a risk of bias assessment was completed for each included RCT using the Cochrane risk-of-bias assessment tool for randomized trials (Higgins et al., 2011). The Cochrane tool explores bias over five domains: selection bias, performance bias, detection bias, attrition bias and reporting bias. There is an additional domain for crossover trials that explores any carryover bias. Each study was judged across all domains and was given an overall rating. The risk of bias assessment was completed independently by three reviewers, ensuring each study was double-rated by two different reviewers. Disagreements were discussed and resolved through consensus.

### Data extraction and analysis

Descriptive data were extracted by three independent reviewers and organised into a table (Table 1), including the type of study (single- or double-blinded RCT), population (disorder, number and whether medication-free) studied, intervention used (type and dose(s) of ketamine, route of administration and frequency of dosing if applicable), control used (details of the inactive or active placebo) and outcome measured (anxiety scale used; anxiety scores at time points measured).

**Table 1.** Study characteristics.

Lead author	Randomised sample size (n)	Blinding	Sample disorder	Medication	Study design	Ketamine (type, dose and route)	Single or repeated dose	Placebo (dose and route)	Primary/secondary measure(s)	Anxiety scale in meta-analysis	Time stamp included in meta-analysis
Abdallah et al. (2022)	Ketamine (0.5 mg/kg): 51Ketamine (0.2 mg/kg): 53Placebo: 54	Double-blinded	PTSD	Concomitant medication	Parallel group	Racemic Intravenous (0.5 mg/kg; 0.2 mg/kg)	Repeated dose	Saline solution Intravenous	PCL-5: primary; CAPS-5: secondary	PCL-5	Subacute (24 h) Sustained (day 7)
Dadabayev et al. (2020)	Ketamine: 11Placebo: 10		PTSD and chronic pain	Concomitant medication	Parallel group	Racemic Intravenous (0.5 mg/kg)	Single dose	Ketorolac 15 mg Intravenous	IES-R: primary	IES-R	Subacute (24 h) Sustained (day 7)
Fallon et al. (2018)	Ketamine: 107Placebo: 107		Cancer-related neuropathic pain	Concomitant medication	Parallel group	Racemic Oral (various doses)	Single dose	Inert placebo (various doses) Oral	HADS: secondary	X	X
Feder et al. (2014)	Ketamine: 22Placebo: 19		PTSD	Concomitant medication	Crossover	Racemic Intravenous (0.5 mg/kg)	Single dose	Midazolam (0.045 mg/kg) Intravenous	IES-R: primary	IES-R*	Subacute (24 h) Sustained (day 7)
Feder et al. (2021)	Ketamine: 15Placebo: 15		PTSD	Concomitant medication	Parallel group	Racemic Intravenous (0.5 mg/kg)	Repeated dose	Midazolam (0.045 mg/kg) Intravenous	CAPS-5: primary; IES-R: secondary	Subacute: IES-R; sustained: CAPS-5	Subacute (24 h) Sustained (14 days)
Lapidus et al. (2014)	Ketamine: 9Placebo: 9		Treatment-resistant depression	Concomitant medication	Crossover	Racemic Intranasal (50 mg)	Single dose	Saline solution Intravenous	HAM-A: secondary	X	X
Murrough et al. (2015)	Ketamine: 12Placebo: 12		Mood disorders with suicidal ideation	Concomitant medication	Parallel group	Racemic Intravenous (0.5 mg/kg)	Single dose	Midazolam (0.045 mg/kg) Intravenous	CAST: secondary	CAST – Anxiety subscale	Subacute (24 h) Sustained (day 7)
Norbury et al. (2021)	Ketamine: 11Placebo: 10		PTSD	Concomitant medication	Parallel group	Racemic Intravenous (0.5 mg/kg)	Repeated dose	Midazolam (0.045 mg/kg) Intravenous	CAPS-5: primary	X	X
Nugent et al. (2019)	Ketamine: 18 (n from personal communication: 33)Placebo: 17 (n from personal communication: 31)		Treatment-resistant depression	Unmedicated	Crossover	Racemic Intravenous (0.5 mg/kg)	Single dose	Saline solution Intravenous	HAM-A: secondary	HAM-A	Acute (230 min) Subacute (24 h) Sustained (day 7)
Pradhan et al. (2017)	Ketamine: 5Placebo: 5		PTSD and treatment-resistant depression comorbidity	Concomitant medication	Crossover	Racemic Intravenous (0.5 mg/kg)	Single dose	Saline solution Intravenous	PCL, CAPS: primary	PCL	Acute (4 h) Subacute (24 h)
Pradhan et al. (2018)	Ketamine: 10Placebo: 10		PTSD	Concomitant medication	Parallel group	Racemic Intravenous (0.5 mg/kg)	Single dose	Saline solution Intravenous	PCL, CAPS: primary	PCL	Subacute (24 h)
Rodriguez et al. (2013)	Ketamine: 8Placebo: 7		OCD	Unmedicated	Crossover	Intravenous (0.5 mg/kg)	Single dose	Saline solution Intravenous	OCD-VAS, Y-BOCS: primary	Acute: OCD-VAS* Sustained: Y-BOCS	Acute (230 min) Sustained (day 7)
Taylor et al. (2018)	Ketamine: 9Placebo: 9		SAD	Concomitant medication	Crossover	Intravenous (0.5 mg/kg)	Single dose	Saline solution Intravenous	LSAS, VAS: primary; STAI-S: secondary	LSAS* – acute and sustained	Acute (3 h) Subacute (24 h) Sustained (day 7)
Zarate et al. (2012)	Ketamine: 7 (n from personal communication: 14)Placebo: 8 (n from personal communication: 12)		Bipolar disorder	Concomitant medication	Crossover	Intravenous (0.5 mg/kg)	Single dose	Saline solution Intravenous	HAM-A, VAS-Anxiety: secondary	HAM-A	Acute (230 min) Subacute (24 h) Sustained (day 7)

Anxiety-VAS: Anxiety-Visual Analogue Scale; CAPS: Clinical-Administered PTSD Scale; CAPS-5: Clinically Administered Posttraumatic Stress Disorder Scale for DSM-5; CAST: Concise Associated Symptoms Tracking scale; HAM-A: Hamilton Anxiety Rating Scale; IES-R: Impact of Events Scale-Revised; LSAS: Liebowitz Social Anxiety Scale; OCD: obsessive-compulsive disorder; OCD-VAS: Obsessive Compulsive Disorder-Visual Analogue Scale; PCL: Posttraumatic Stress Disorder Checklist; PCL-5: Posttraumatic Stress Disorder Checklist for DSM-5; PTSD: post-traumatic stress disorder; SAD: social anxiety disorder; STAI-S, State-Trait Anxiety Subscale; Y-BOCS: Yale-Brown Obsessive Compulsive Scale.

* Indicates that the value included in meta-analysis was estimated.

Numerical data were extracted directly from published papers (when available), from direct communication with authors, or from estimating graph values using WebPlotDigitizer (Rohatgi, 2015) if needed. Extracted numerical data were then compiled using Review Manager (Review Manager [RevMan] version 5.4, 2020), and RevMan was used to generate forest plots of the standard mean differences (SMDs) in anxiety scores between groups receiving ketamine versus placebo. Separate analyses were carried out on three time points: less than 12 h post-administration (acute), 24 h post-administration (subacute) and 7–14 days post-administration (sustained). A time point beyond 14 days was also qualitatively assessed. To maximise comparability, when a study had multiple time points which fell under one of our pre-specified ranges, the modal time point that was available across studies was included (unless a study only had data available at a different time point). In the case of multiple anxiety outcomes, the primary outcome as defined by the study was used. In crossover trials where a carryover effect was identified by the authors, data from the first arm only were included in the meta-analysis.

Data were pooled across studies to conduct exploratory analyses of the correlation between improvements in anxiety scores post-ketamine and (1) improvements in depression scores post-ketamine and (2) peak Clinician-Administered Dissociative States Scale (CADSS) scores using linear regressions in R (version 3.5.3).

## Results

**Figure 1.**
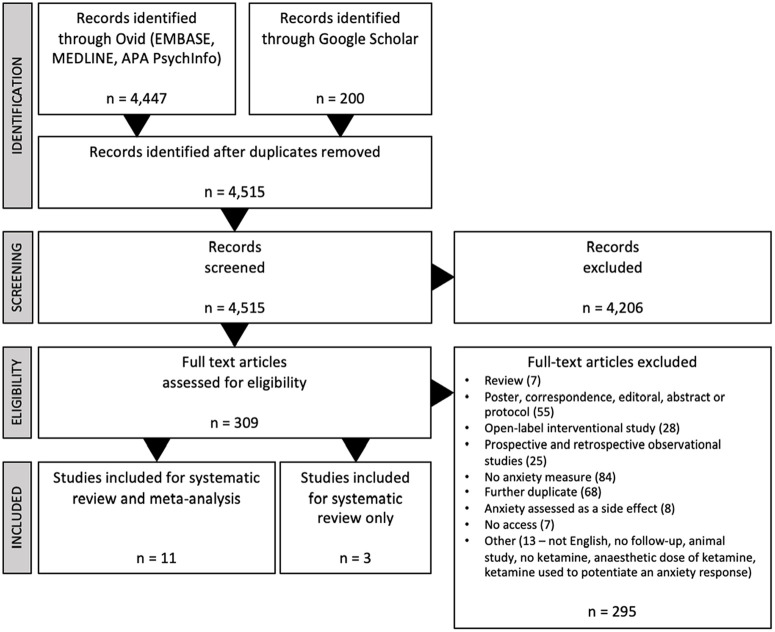
PRISMA flow chart.

Results from the systematic review and meta-analysis article search are summarised in the PRISMA flowchart (Figure 1). The combined searches generated 4647 records, leaving 4515 once duplicates were removed. After initial screening, 309 articles were included in the full-text review. Of these, 295 were deemed ineligible, and 14 RCTs were included in the qualitative systematic review. Due to missing and inaccessible data, 3 were excluded from quantitative analysis, meaning 11 articles were included in the meta-analysis.

### Study characteristics and risk of bias

**Figure 2.**
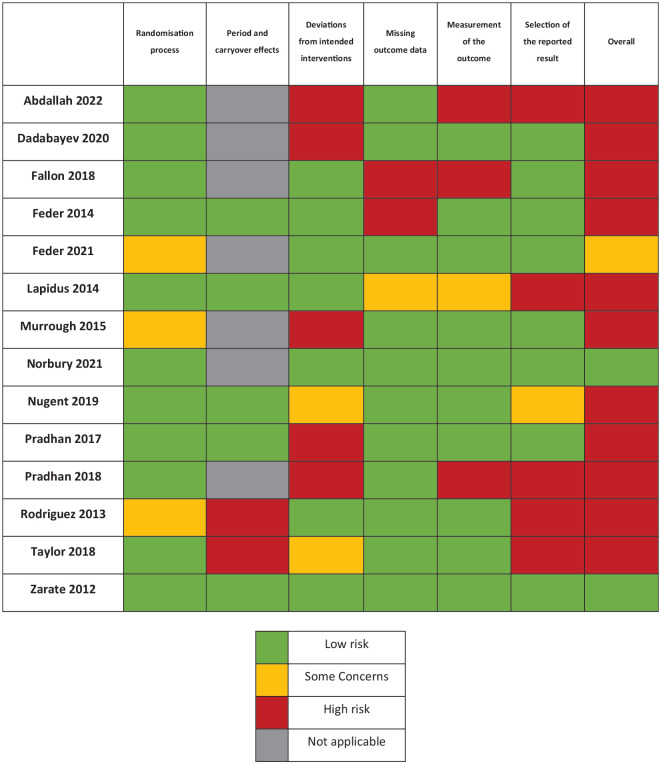
Cochrane risk of bias table for included studies (*k* = 14).

Parameters for each study, including design, sample sizes, ketamine dosing, control dosing, and outcome measures, are reported in Table 1. Results of the Cochrane risk of bias analysis revealed that all but two studies had an overall rating of some concerns or high risk of bias (Figure 2). The most common domains of concern were deviations from intended interventions, selective reporting of results and carryover effects in two of the seven studies with crossover designs.

The subsequent sections present analyses divided into ketamine’s acute response (less than 12 h; *k* = 6), subacute response (24 h; *k* = 10), sustained response (7–14 days; *k* = 9) and responses beyond 14 days (*k* = 4). For a detailed summary of significant and non-significant findings of each study, see Supplemental Table 1.

### Acute (<12 h)

Seven studies measured anxiety at one or more time points <12 h after ketamine administration. Two of these studies did not report their findings (Lapidus et al., 2014; Pradhan et al., 2018). Of the rest, three reported that ketamine reduced anxiety significantly compared to placebo at one time point at least (Nugent et al., 2019; Rodriguez et al., 2013; Zarate et al., 2012), and two reported non-significance compared to placebo (Pradhan et al., 2017; Taylor et al., 2018).

All three significant studies used intravenous ketamine. Zarate et al. (2012) (*n* = 15) and Nugent et al. (2019) (*n* = 35) explored anxiolytic effects in mood disorder participants, and Rodriguez et al. (2013) (*n* = 15) explored effects in patients with OCD. Zarate et al. (2012) found significant improvements in the Visual Analogue Scale for anxiety (VAS-Anxiety; Aitken, 1969) starting at 40 min post-infusion, which remained significant at 80, 110 and 230 min. Nugent et al. (2019) found significant improvements in Hamilton Anxiety Scale (HAM-A; Hamilton, 1959) scores at 230 min, but not 40 min. Similarly, Rodriguez et al. (2013) found significant improvements in the VAS for OCD (OCD-VAS; Rodriguez et al., 2011) scores at 230 min, but not at 90 min and 110 min.

**Figure 3.**
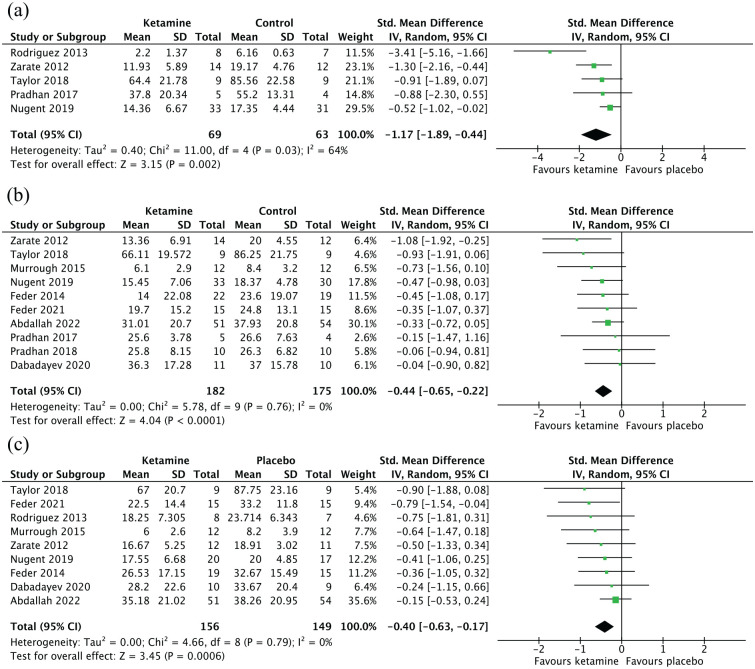
Forest plots. Left favours ketamine (ketamine reduced anxiety scores compared to placebo). Error bars represent 95% confidence intervals: (a) acute time point (<12 h), (b) subacute time point (24 h) and (c) sustained time point (7–14 days).

For the meta-analysis, group-level data were obtained for all five studies with reported data taken between 3 and 4 h post-administration (Figure 3(a)). This meta-analysis included 69 patients who received ketamine and 63 who received placebo. SMDs were calculated for each study, as well as an overall SMD for the meta-analysis, which was significant in favour of ketamine compared to placebo (SMD: −1.17, 95% confidence interval (CI) [−1.89, −0.44], *p* < 0.01). There was significant heterogeneity among the studies (*I*^2^ = 64%, *p* = 0.03), and so a sensitivity analysis was performed excluding Rodriguez et al. (2013), which was the only study to use a single-item VAS rather than a multi-item measure to measure anxiety symptoms. We found that anxiety scores were still significantly lower in the ketamine group compared to the placebo group (SMD: −0.75, 95% CI [−1.14, −0.37], *p* < 0.01), and that heterogeneity had been eliminated (*I*^2^ = 0%, *p* = 0.47), suggesting that heterogeneity arose from inclusion of this study.

### Subacute (24 h)

Most studies included in this review reported findings at 24 h post-ketamine administration (*k* = 10). Of these studies, four reported a significant effect of ketamine in reducing anxiety symptoms compared to placebo: three using the HAM-A scale in mood disorder patients (Lapidus et al., 2014; Nugent et al., 2019; Zarate et al., 2012) and one using the Impact of Event Scale – Revised (IES-R; Weiss & Marmar, 1997) in PTSD patients (Feder et al., 2014). All four studies used a crossover design.

Lapidus et al. (2014) (*n* = 20) and Nugent et al. (2019) (*n* = 60) found intranasal and intravenous ketamine treatments to be more effective in reducing HAM-A scores in MDD patients than placebo at 24 h post-administration. Similarly, Zarate et al. (2012) (*n* = 15) found intravenous ketamine to be superior to placebo at symptom reduction using this scale at 24 h in bipolar disorder patients. Lastly, Feder et al. (2014) (*n* = 41) reported significant drug group differences at 24 h on the IES-R when comparing the efficacy of 0.5 mg/kg ketamine with an active placebo, midazolam (0.045 mg/kg) in 41 patients with chronic PTSD.

Results of the meta-analysis of 10 studies with available group-level data (Figure 3(b)) showed that there was a significant difference in anxiety scores between the ketamine (*n* = 179) and placebo (*n* = 178) groups (SMD: −0.44, 95% CI: [−0.65, −0.22], *p* < 0.01). No significant heterogeneity was found (*I*^2^ = 0%, *p* = 0.76).

### Sustained (7–14 days)

There were mixed findings from the nine studies which looked at time points between 7 and 14 days post-infusion – four reported significant findings (Feder et al., 2021; Rodriguez et al., 2013; Taylor et al., 2018; Zarate et al., 2012), and three reported no significance (Abdallah et al., 2022; Feder et al., 2014; Nugent et al., 2019). It is of note that results from one study using multiple ketamine dosages (Abdallah et al., 2022) imply the potential of lower doses of ketamine to exhibit efficacy in anxiolysis (0.2 mg/kg, rather than 0.5 mg/kg). One study reported anxiety measures but did not analyse them statistically (Murrough et al., 2015).

Feder et al. (2021) (*n* = 30) used scores from the Clinician-Administered PTSD Scale for DSM-5 (CAPS-5; Weathers et al., 2018) to assess symptom severity 1 week into treatment with multiple doses of intravenous ketamine (measured after fourth infusion) and at the end of treatment at 2 weeks. Analysis revealed significantly lower total scores in the ketamine group compared to the midazolam group at 1 week post-first infusion, which was sustained at 2 weeks post-first infusion. Rodriguez et al. (2013) (*n* = 15) found that patients with OCD who received a single infusion of ketamine reported significantly lower means on the OCD-VAS at 7 days post-infusion. Meanwhile, Taylor et al. (2018) (*n* = 18) found that, starting at 10 days post-infusion, participants with SAD receiving ketamine demonstrated significantly greater reductions in overall Liebowitz Social Anxiety Scale (LSAS; Heimberg et al., 1999) scores compared to those receiving placebo.

Both Zarate et al. (2012) and Murrough et al. (2015) explored ketamine’s efficacy in patients diagnosed with mood disorders. Zarate et al. (2012) reported significantly lower scores in subjects who had received ketamine as opposed to placebo on the VAS-Anxiety scale at days 7 and 14 post-ketamine administration. In patients with mood disorders and clinically significant suicidal ideation, Murrough et al. (2015) (*n* = 24) found that mean Concise Associated Symptoms Tracking scale (CAST; Trivedi et al., 2011) subscale scores pertaining to anxiety (irritability, anxiety and panic) were numerically lower in the ketamine compared to midazolam group on each subscale at 7 days, but the researchers did not analyse the CAST subscale scores statistically.

The meta-analysis included nine studies for which data were available (Figure 3(c)) and included 157 patients who had received ketamine and 150 patients who had received placebo. Analysis revealed that mean anxiety scores were significantly lower in the ketamine group compared to those in the placebo group (SMD: −0.40, 95% CI [−0.63, −0.17], *p* < 0.01). There was no significant heterogeneity among studies (*I*^2^ = 0%, *p* = 0.79).

### Effects beyond 14 days

Of the four studies which explored the efficacy of ketamine beyond 2 weeks, two studies reported significant treatment group differences (Norbury et al., 2021; Pradhan et al., 2018), whilst two reported non-significance (Fallon et al., 2018; Pradhan et al., 2017).

A repeated dose study carried out by Norbury et al. (2021) found CAPS-5 scores to be significantly lower at 16 days post-first dose in PTSD patients who had received ketamine than those who had received midazolam. Results revealed a significant session-by-drug interaction on CAPS-5 scores (*F*_1,57_ = 6.58, *p* = 0.013). Also exploring a prolonged treatment response were the Pradhan studies (Pradhan et al., 2017, 2018), which measured Clinical Administered PTSD Scale (CAPS; Blake et al., 1995) and PTSD Checklist for DSM-IV (PCL; Weathers et al., 2013) scores weekly until relapse. In the 2017 study, patients receiving ketamine had a more sustained response (33 ± 22.98 days) than those who received placebo (25 ± 16.8 days), though this difference was non-significant (*p* = 0.545). In the 2018 study, the difference in length of response demonstrated a similar pattern (34.44 ± 19.12 days in the ketamine group and 16.50 ± 11.39 in the placebo group); however, the difference was significant (*p* = 0.022).

### Correlation between improvement in anxiety and depression scores

**Figure 4.**
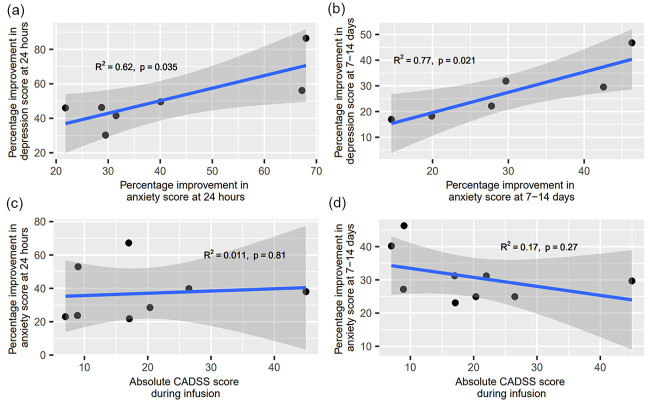
Exploratory analysis of the correlation between ketamine’s (a) anxiolytic and antidepressant effects at 24 h (significant positive correlation; *R*^2^ = 0.621, *p* = 0.035), (b) anxiolytic and antidepressant effects at 7–14 days (significant positive correlation; *R*^2^ = 0.773, *p* = 0.021), (c) anxiolytic effects at 24 h and peak CADSS scores (no correlation; *R*^2^ = 0.011, *p* = 0.808), and (d) anxiolytic effects at 7–14 days and peak CADSS scores (no correlation; *R*^2^ = 0.171, *p* = 0.268). CADSS: clinician-administered dissociative states scale.

Seven studies provided depression and anxiety data at the subacute time point and six studies provided this data at the sustained time point, leading to 182 and 175 participants included in each analysis, respectively. There was a significant correlation between percentage improvement in anxiety and depression scores at the subacute time point (*R*^2^ = 0.621, *p* = 0.035, Figure 4(a)) and at the sustained time point (*R*^2^ = 0.773, *p* = 0.021; Figure 4(b)).

### Correlation between improvement in anxiety and peak dissociation

We explored the relationship between peak dissociation and anxiety scores by correlating peak CADSS (Bremner et al., 1998) scores with percentage improvement in anxiety at the subacute and sustained time points. All peak CADSS scores were during infusion, except for Taylor et al. (2018), which was measured at 1-h post-ketamine administration.

At 24 h post-administration, data from 222 participants from 8 studies were pooled to be included in the analysis. Results revealed no significant correlation between peak level of dissociation and improvement in anxiety symptoms (*R*^2^ = 0.011, *p* = 0.808; Figure 4(c)). Similarly, at 7–14 days post-administration, where data from 237 participants from 9 studies were pooled, there was no significant correlation between the two scores (*R*^2^ = 0.171, *p* = 0.268; Figure 4(d)).

## Discussion

This is the first transdiagnostic systematic review and meta-analysis of RCTs to assess the temporal profile of ketamine’s anxiolytic effects after a single dose. Of the 14 studies included in the systematic review and meta-analysis, seven studies assessed ketamine’s anxiolytic effects in PTSD; five in mood disorders; two in anxiety disorders; and two in either chronic or cancer-related pain. Results suggest that ketamine is an efficacious treatment for anxiety symptoms across this range of settings. Ketamine’s anxiolytic effect typically emerged after 3–4 h and continued to be significantly superior relative to placebo at 24 h and 7–14 days post-administration. Our findings suggest that single ketamine infusions could therefore offer both rapid and sustained improvement in anxiety. This work builds on previous systematic reviews and meta-analyses supporting ketamine’s use in anxiety disorders (Banov et al., 2020; Tully et al., 2022; Whittaker et al., 2021).

Seven studies reported data <12 h after administration. Our results at this acute time point support the conclusions made by both preclinical and clinical studies that ketamine may result in rapid reductions in anxiety (Glue et al., 2017; Riehl et al., 2011). One hypothesised mechanism of action is a ‘glutamate surge’ in the prefrontal cortex, through blockade of NMDA receptors on pre-synaptic GABAergic inhibitory interneurons (Alexander and Young, 2022). The earliest significant effect was reported by Zarate et al. (2012) at 40 min post-infusion with the majority of studies measuring anxiety symptoms at this time point reporting a significant effect at 3–4 h. This corroborates the finding by Glue et al. (2017) in an open-label study, where ketamine administration led to marked improvements in anxiety scores at 1 h post-dose in patients with generalised anxiety disorder and/or SAD. Heterogeneity was significant at this time point but was eliminated in a sensitivity analysis excluding the one study using VAS to measure anxiety symptoms (Rodriguez et al., 2013). This possibly reflects the higher sensitivity of VAS compared to multi-item scales. In this sensitivity analysis, ketamine still significantly improved anxiety scores compared to placebo.

In all, 10 studies reported data at 24 h post-administration – the modal time point in the meta-analysis – where ketamine again had a beneficial effect compared to placebo. Although at 24 h post-administration ketamine has been eliminated from circulation, the maintained therapeutic effects may be related to initial changes in AMPA receptor trafficking, together with neuroplastic effects mediated by brain-derived neurotrophic factor and tyrosine kinase receptor signaling (Alexander and Young, 2022). Our findings at this time point align with the literature illustrating the time course of ketamine’s antidepressant effects (Phillips et al., 2019), and also with results from responder data, which provides additional insight into whether a drug provides clinically meaningful symptom relief. For example, Glue and colleagues showed that patients with SAD and GAD show a higher likelihood of response at 24 h after ketamine compared to midazolam (Glue et al., 2020).

Nine studies reported data at 7–14 days after administration. The sustained time point had the most RCTs report significant drug differences out of all time points. This suggests that ketamine has anxiolytic effects which last through 1–2 weeks post-treatment. At this time point, ketamine’s effect is mediated by neuroplastic changes mediated by changes in gene expression and ultimately resulting in synaptogenesis (Alexander and Young, 2022). Our findings parallel the literature exploring ketamine’s efficacy in depression, which has found ketamine to have a sustained antidepressant effect lasting 7–14 days after a single dose (Coyle and Laws, 2015).

We endeavoured to examine any anxiolytic effects beyond 14 days. However, very few studies met our inclusion criteria (*n* = 4). From the included studies, there is evidence that ketamine continues to be superior to placebo beyond 2 weeks post-administration, with one study finding evidence of an anxiolytic effect up to 1 month after a single dose (Pradhan et al., 2018). Coupling these results with the optimism in findings from earlier time points, future studies should further explore ketamine’s sustained anxiolytic efficacy beyond that of 2 weeks.

To date, most research into the psychiatric utility of ketamine has focused on depression. We conducted an exploratory analysis into the relationship between depression and anxiety improvements at the subacute and sustained time point. If the anxiolytic and antidepressant effects of ketamine share similar mechanisms, one might expect to see a strong correlation between the improvements in both symptoms. Results revealed a significant correlation between mean percentage improvements in depression and anxiety at both the subacute and sustained time points. Our exploratory analysis was based on pooled data from multiple studies, and so could not control for any covariant effects of mood changes on ketamine’s anxiolytic effects. This should be addressed in future research to understand the totality of the medication’s therapeutic effects. Additionally, future work could explore whether the same or distinct neural circuits are related to ketamine’s effects on distinct symptom clusters.

We also analysed whether there was any link between ketamine’s anxiolytic effect and its peak dissociative effects as measured using the CADSS, at both the subacute and sustained time points. Research on the relationship between dissociation and therapeutic outcomes of ketamine treatment is equivocal. There are documented concerns that ketamine’s dissociative effects may negatively impact anxiety outcomes (Carlson et al., 2012; Coutinho et al., 2016; Schönenberg et al., 2005, 2008). Conversely, there is the suggestion that the degree of dissociation is important in ketamine’s therapeutic effects, and that greater levels of dissociation may lead to greater improvements in depression symptoms (Correia-Melo et al., 2017; Luckenbaugh et al., 2014; Sos et al., 2013). Finally, other work has concluded no relationship between peak dissociation and therapeutic effect (Ballard and Zarate, 2020; Berman et al., 2000; Fava et al., 2020; Lapidus et al., 2014; Mathai et al., 2023; Włodarczyk et al., 2021). We did not have enough data to analyse the relationship between dissociation and anxiety at <12 h (four data points). At the subacute and sustained time points, our analysis showed no significant relationship between the two, suggesting that ketamine’s anxiolytic effects are independent of its dissociative effects. However, this exploratory analysis was based on relatively few data points of pooled means, and so further work could explore this relationship more specifically.

Several limitations of our study are of note. First is the high risk of bias in most of the included studies. Nine out of the 11 studies were reported as having high risk of bias, mainly because of unblinding of patients and outcome assessors, or selective reporting of data from secondary measures. The prevalence of unblinding in the included studies speaks to the difficulty in achieving effective blinding in studies exploring ketamine’s efficacy. Only five studies included an active placebo, and future research would benefit from the use of active placebos as controls.

A second limitation is the presence of moderate heterogeneity in the meta-analysis of data at the acute time point (*I*^2^ = 64%) which implies clinical and/or methodological diversity in the included studies and implicates our conclusions. This may be due to differences in measurement tools, as suggested by the elimination of heterogeneity in our sensitivity analysis excluding Rodriguez et al. (2013), which was the only study to use a single-item versus multi-item score to measure anxiety. Other contributing factors may include low sample sizes (Inthout et al., 2015), differences in precise time points and differences in disorders (though the latter is inherent in prioritising a transdiagnostic approach). While heterogeneity was low in the other two time points (*I*^2^ = 0% in subacute and sustained analyses), the potential for issues in comparability should not be ignored. Future work should aim for larger sample sizes and to carry out analyses to explore the influence of sex, age and other demographics.

Third, our meta-analyses consisted of findings from parallel arm and crossover studies. When carryover effects were found in crossover trials, data were limited to exclusively the first phase of the study; if not, collapsed data from both phases were used. It is possible that this variability introduced bias into our analysis. Finally, although this review intended to include studies assessing ketamine’s anxiolytic efficacy in settings such as chronic pain and palliative care, only one study in this context was eligible for inclusion (Dadabayev et al., 2020).

## Conclusion

Our meta-analysis demonstrates ketamine’s anxiolytic effects emerged rapidly at 3–4 h post-administration and persisted for up to 2 weeks. This study is the first review to determine the efficacy of ketamine for treatment of anxiety symptoms across multiple time points using a transdiagnostic approach. By limiting our review to randomised control trials, we included data from the highest quality of medical evidence and were able to mitigate the effects of confounds and bias as much as possible, whilst identifying areas of uncertainty in the literature. Future RCTs should explore anxiolytic effects after repeated dosing and assess the effects of different doses of ketamine on anxiety symptoms. This will help to identify the optimum administration pattern for the use of ketamine as an anxiolytic agent in clinical practice.

## Supplemental Material

sj-docx-1-jop-10.1177_02698811231161627 – Supplemental material for A transdiagnostic systematic review and meta-analysis of ketamine’s anxiolytic effects Supplemental material, sj-docx-1-jop-10.1177_02698811231161627 for A transdiagnostic systematic review and meta-analysis of ketamine’s anxiolytic effects by Hannah Hartland, Kimia Mahdavi, Luke A Jelen, Rebecca Strawbridge, Allan H Young and Laith Alexander in Journal of Psychopharmacology
